# Radical cystectomy highlighted in International Brazilian Journal of Urology

**DOI:** 10.1590/S1677-5538.IBJU.2022.01.01

**Published:** 2021-12-21

**Affiliations:** 1 Universidade do Estado do Rio de Janeiro - Uerj Unidade de Pesquisa Urogenital Rio de Janeiro RJ Brasil Unidade de Pesquisa Urogenital - Universidade do Estado do Rio de Janeiro - Uerj, Rio de Janeiro, RJ, Brasil; 2 Hospital Federal da Lagoa Serviço de Urologia Rio de Janeiro RJ Brasil Serviço de Urologia, Hospital Federal da Lagoa, Rio de Janeiro, RJ, Brasil

The January-February number of Int Braz J Urol, the 14th under my supervision, presents original contributions with a lot of interesting papers in different fields: Prostate Cancer, Male Infertility, Renal Cell Carcinoma, Urinary Stones, Bladder Cancer, BPH, Neurogenic Bladder, Undescended Testis, Stress Urinary Incontinence, Posterior Urethral valves, Robotic Prostatectomy, Wilms tumor and Covid-19 in Urology. The papers came from many different countries such as Brazil, USA, Italy, Argentina, Turkey, Egypt, Saudi Arab, Germany, Denmark and India, and as usual the editor's comment highlights some of them.

In the present issue we present two important papers about bladder cancer and radical cystectomy. Dr. Korkes and colleagues from Brazil performed in page 18 (1) a systematic review comparing ileal conduit (IC) and cutaneous ureterostomy (CU) urinary diversions (UD) in terms of perioperative, functional, and oncological outcomes of high-risk elderly patients treated with radical cystectomy (RC) and concluded CU seems to be a safe alternative for the elderly and more frail patients. It is associated with faster surgery, less blood loss, lower transfusion rates, a lower necessity of intensive care, and shorter hospital stay. Dr. Eismann and colleagues from Germany performed in page 89 (2) a nice study about the characteristics of Contrast-enhanced CT scan to predict lymph node ratio (LNR) and its impact on survival in patients with bladder cancer undergoing radical cystectomy (RC) and concluded that LN size >15mm significantly correlated with higher LNR and identification of increased loco-regional LN was associated with worse survival. The editor in chief would like to highlight the following works too:

Dr. Benzi and collegues from Brazil presented in page 8 (3) a nice descritive review about the translational and anatomic aspects of testicular vascularization applied to Fowler-Stephens surgery for high undescended testis and concluded that the laparoscopic transection of the testicular vessels by dividing the spermatic vessels is safe in patients with high abdominal testis due to the great collateral vascular supply between testicular, vasal and cremasteric arteries; also, two-stage Fowler-Stephens orchiopexy appears to carry a higher rate of success than the single stage approach. This important paper is the cover in this edition.

Dr. Sager and collegues from Argentina e Brazil presented in page 31 (4) a important update about the management of neurogenic bladder dysfunction in children and concluded that clean intermittent catheterization (CIC) should be implemented during the first days of life, and antimuscarinic drugs should be indicated upon results of urodynamic studies. When the patient becomes refractory to first-line therapy, receptor-selective pharmacotherapy is available nowadays, which leads to a reduction in reconstructive procedures, such as augmentation cystoplasty.

Dr. Turkoglu and collegues from Turkey presented in page 70 (5) a nice study about the role of transperineal ultrasound in the evaluation of stress urinary incontinence cases and concluded that

Dr. Sarhan and collegues from Egypt presented in page 78 (6) a important study about the bladder function in children with posterior urethral valves (PUVs): impact of antenatal versus postnatal diagnosis and concluded that compared to delayed intervention, primary ablation of PUVs during the early neonatal life possibly provides the optimum chance to have optimum renal function without impact on bladder function.

Dr. Dubeux and collegues from Brazil presented in page 110 (7) a nice sudy about the practical evaluation of the R.E.N.A.L. score system (RS) in 150 laparoscopic nephron sparing surgeries and concluded that RS system is a good way to predict adverse outcomes in nephron sparring surgery, especially in cases over 7. Further studies should focus on robotic approach and patient's characteristics other than the masses’ aspects.

Dr. Humaidan and collegues from Denmark presented in page 131 (8) a nice study about the combined effect of lifestyle intervention and antioxidant therapy on sperm DNA fragmentation and seminal oxidative stress in IVF patients and concluded that a 3-month lifestyle intervention program combined with antioxidant therapy reduced DNA fragmentation index (DFI) in infertile men with elevated Sperm DNA fragmentation (SDF) and a history of failed IVF/ICSI. A personalized lifestyle and antioxidant intervention could improve fertility of subfertile couples through a reduction in DFI, albeit controlled trials evaluating reproductive outcomes are needed before firm conclusions can be made.

The Editor-in-chief expects everyone to enjoy reading.
